# Spontaneous Spinal Epidural Hematoma Mimicking Stroke in a Young Patient: A Case Report

**DOI:** 10.7759/cureus.83994

**Published:** 2025-05-12

**Authors:** María F Castelo-Pablos, Irene Gómez-Oropeza, Daniela Deustúa-Hernández, Obet Canela-Calderon, Juan N Arriada-Mendicoa

**Affiliations:** 1 Department of Neurosurgery, National Institute of Neurology and Neurosurgery, Mexico City, MEX; 2 Department of Neurology, National Institute of Neurology and Neurosurgery, Mexico City, MEX

**Keywords:** case report, cervical pain, hemiparesis, spinal cord compression, stroke mimic

## Abstract

Spontaneous spinal epidural hematoma (SSEH) is a rare but serious neurological emergency characterized by the accumulation of blood in the epidural space without an identifiable cause. It typically presents with sudden-onset neck or back pain, followed by motor and sensory deficits. Although uncommon, SSEH can mimic ischemic stroke when presenting as hemiparesis, often leading to diagnostic delays. We report the case of a 35-year-old male patient who developed sudden-onset cervical pain, followed by right-sided hemiparesis and sensory deficits. Due to focal neurological signs, an acute ischemic stroke was initially suspected; however, brain CT was unremarkable. Given the inconclusive findings, a cervicothoracic MRI was performed, which revealed an epidural mass extending from C3 to T2, consistent with SSEH. Surgical decompression confirmed a subacute epidural hematoma with no identifiable source of bleeding. The patient achieved complete neurological recovery within one month of surgery. This case highlights the importance of including SSEH in the differential diagnosis of acute hemiparesis, particularly when preceded by severe neck or back pain and without cranial nerve involvement. It also emphasizes the need to consider spinal imaging when initial brain CT is inconclusive in patients presenting with acute neurological deficits. Early recognition of these clinical features is crucial, as prompt surgical intervention significantly improves neurological outcomes and reduces the risk of long-term disability.

## Introduction

Spontaneous spinal epidural hematoma (SSEH) is a rare but serious neurological condition defined as the accumulation of blood in the epidural space in the absence of preceding trauma or an identifiable underlying cause [1]. It typically presents with sudden-onset motor deficits, such as paraparesis or quadriparesis, accompanied by severe neck or back pain and sensory disturbances. However, in extremely rare cases, SSEH may manifest as hemiparesis, mimicking acute ischemic stroke and potentially leading to misdiagnosis and treatment delays [2,3].

We report the case of a previously healthy young man who developed sudden cervical pain, followed by right-sided hemiparesis, initially prompting stroke code activation. This case highlights the importance of considering SSEH in the differential diagnosis of patients presenting with acute spinal pain and neurological deficits, even in the absence of identifiable risk factors. This report was prepared in accordance with the CARE Guidelines.

## Case presentation

A 35-year-old right-handed man with no significant medical history, including no use of oral anticoagulants, recent trauma, or family history of bleeding disorders, presented to the emergency department with sudden-onset cervical pain followed by neurological deficits.

Two days before admission, the patient experienced sudden, oppressive cervical pain radiating to his back, initially rated 6/10 on the Numeric Pain Rating Scale. The pain persisted for four hours before spontaneously decreasing to 4/10, allowing him to sleep. The following day, it remained at 3/10 but temporarily improved with physical activity. However, later that day, it abruptly worsened to 9/10 and was accompanied by right-sided paresthesia, hemiparesis, and restricted cervical mobility. Upon arrival at the emergency department, a stroke code was activated due to the acute onset of focal neurological deficits.

Clinical examination

Vital signs, laboratory tests, and coagulation parameters were within normal limits. Neurological examination revealed right-sided hemiparesis, with muscle strength graded 4/5 in the upper limb and 3/5 in the lower limb according to the Medical Research Council (MRC) Scale. Sensory examination showed right-sided hypoesthesia. Deep tendon reflexes were hyperreflexic (3+), and the patient exhibited a right extensor plantar response. He also demonstrated leftward lateropulsion while walking and cervical pain that worsened with minimal movement.

Neuroimaging findings

**Figure 1:**
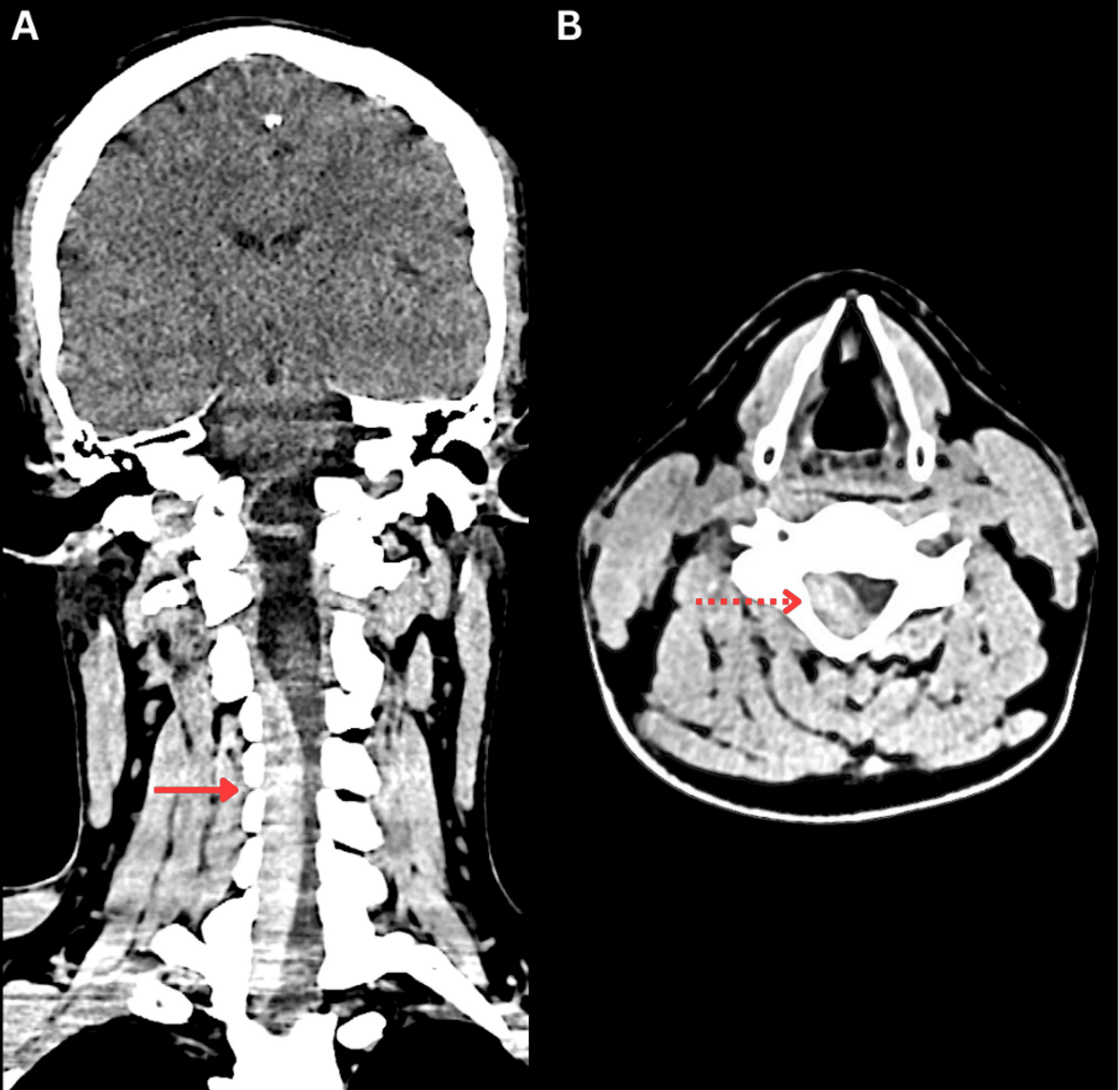
Non-contrast brain and cervicothoracic CT scan showing an epidural hematoma causing spinal cord compression. (A) Coronal plane: hyperdense epidural collection extending from C3 to T2 (solid red arrow). (B) Axial plane at C6: anterior displacement of the spinal cord (dashed red arrow).

A non-contrast brain CT scan showed no evidence of hemorrhage or early signs of ischemia, with an Alberta Stroke Program Early CT Score (ASPECTS) of 10. A cervicothoracic CT scan revealed an elongated hyperdense lesion relative to the spinal cord and surrounding soft tissues, located in the posterior epidural space and extending from the C3 to T2 vertebral levels, causing spinal cord compression and raising suspicion of an epidural hematoma (Figure 1).

**Figure 2:**
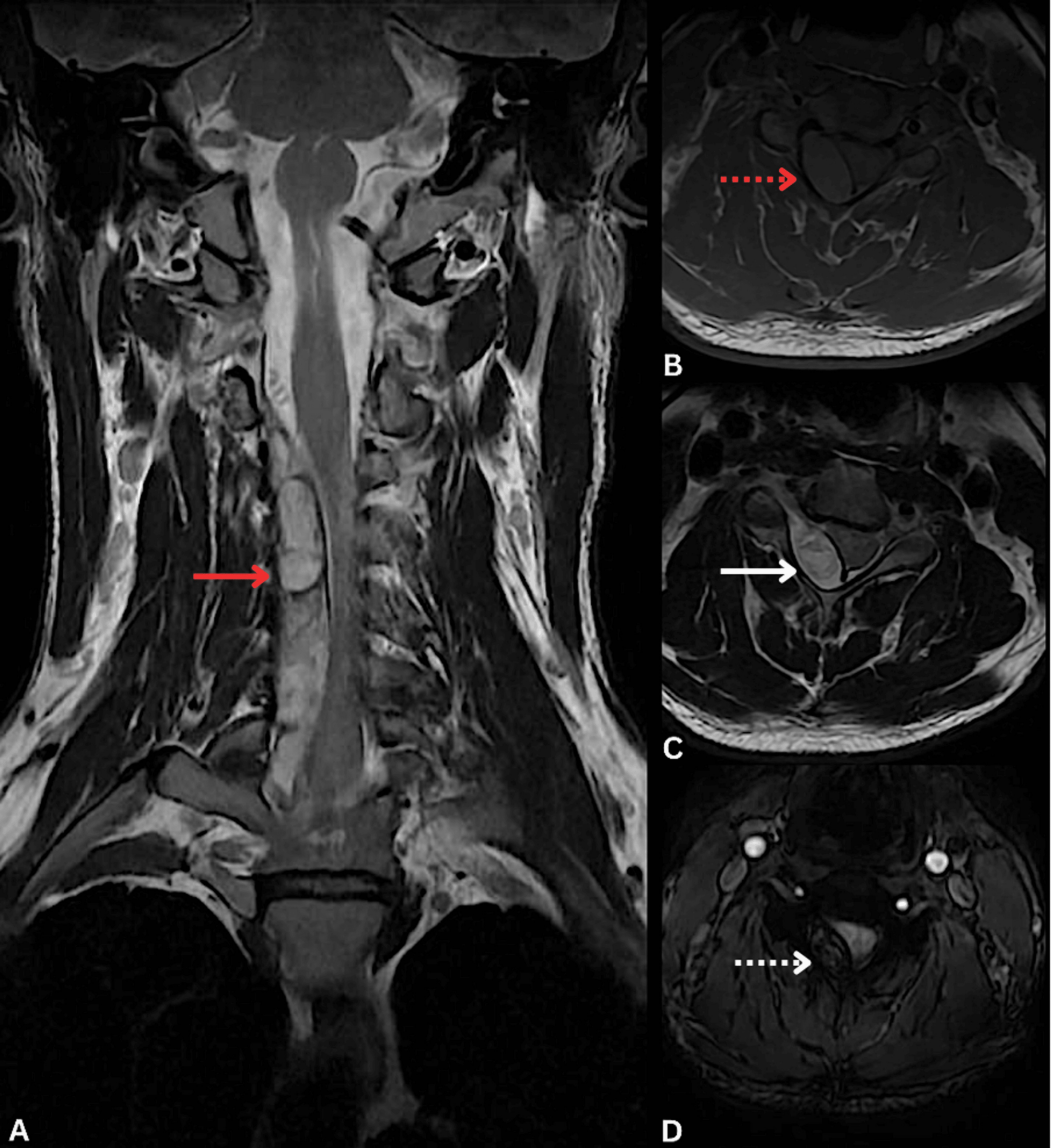
Contrast-enhanced cervical MRI showing an epidural hematoma extending from C3 to T2, causing spinal cord compression. (A) Coronal T2-weighted image: hyperintense epidural lesion (solid red arrow). (B) Axial T1-weighted image: isointense signal (dashed red arrow). (C) Axial T2-weighted image: hyperintense signal (solid white arrow). (D) Axial susceptibility-weighted image (SWI): peripheral hypointense rim with central hypointensity (dashed white arrow).

Subsequent contrast-enhanced cervical magnetic resonance imaging (MRI) confirmed a well-defined epidural mass extending from C3 to T2. The lesion appeared isointense to the spinal cord on T1-weighted images, predominantly hyperintense on T2-weighted images, and showed no post-contrast enhancement. Susceptibility-weighted images (SWI) revealed a peripheral hypointense rim with a central hypointensity, consistent with the presence of blood products (Figure 2). The hematoma caused significant spinal cord compression. It measured 8.1 centimeters in length, with a maximum thickness of 1 centimeter at C5 level. Given these findings, a neurosurgical consultation was requested for definitive surgical management of the SSEH.

Surgical management

**Figure 3:**
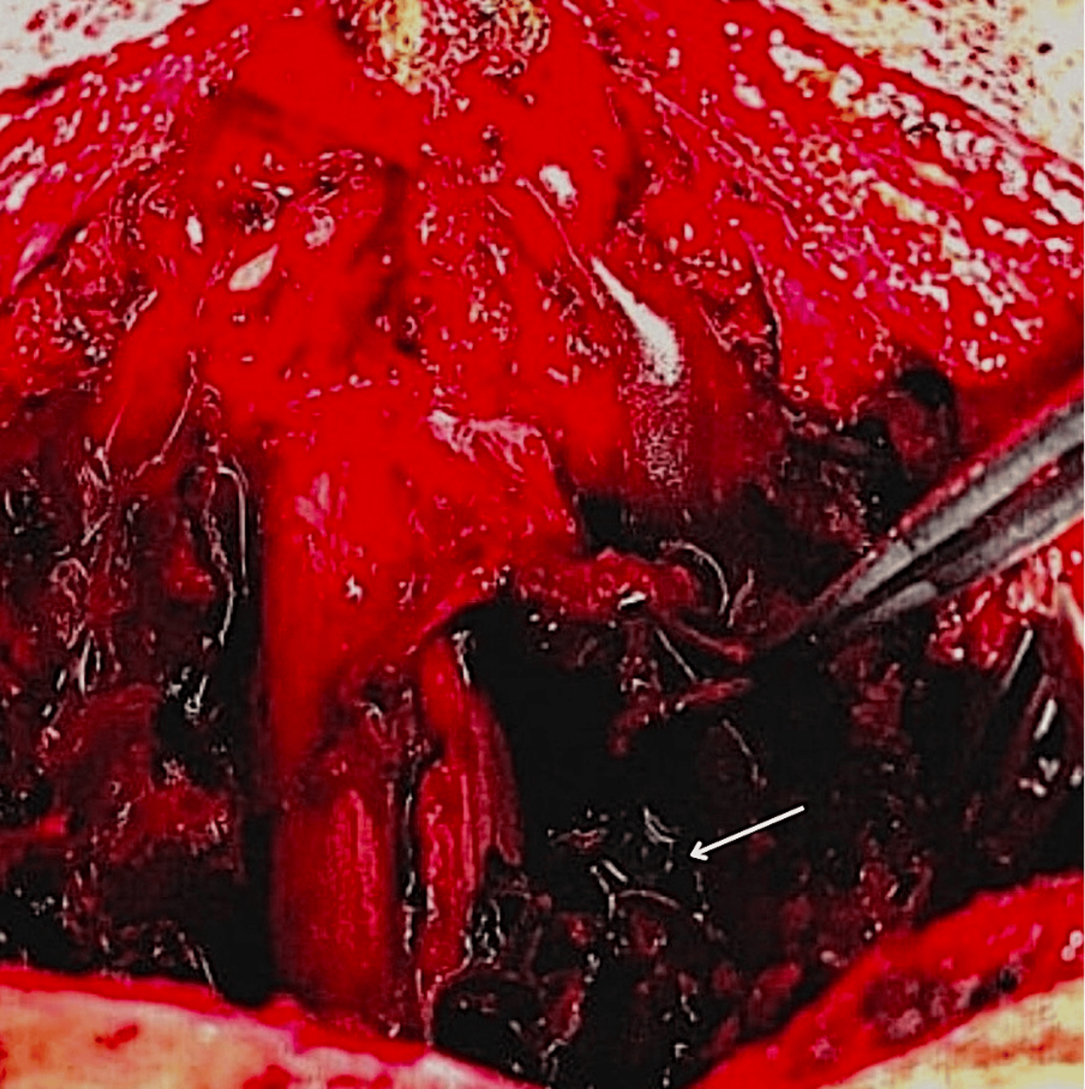
Intraoperative view of the cervical spine after C3-C7 laminectomy, showing a well-organized subacute epidural hematoma (white arrow).

Surgical decompression was performed approximately 32 hours after the onset of neurological deficits. A laminectomy extending from C3 to C7 revealed a well-organized subacute epidural hematoma with no identifiable source of bleeding (Figure 3). The hematoma was successfully evacuated, and the procedure was completed without complications.

Postoperative course

Neurological improvement was evident within two days after surgery, with complete recovery confirmed on examination, including full gait stabilization, normal deep tendon reflexes, and muscle strength graded 5/5 on the MRC Scale in all extremities. During his physical medicine and rehabilitation evaluation on postoperative day 10, he presented with bilateral muscle spasm involving the trapezius, levator scapulae, and splenius muscles, as well as the right sternocleidomastoid. Range of motion was limited due to pain; however, no motor deficits were observed, and both strength and sensation were fully preserved. The patient continued with physical therapy, and at the one-month follow-up, complete resolution of all symptoms was confirmed.

## Discussion

SSEH is an uncommon but potentially severe neurological condition characterized by the accumulation of blood in the epidural space without an identifiable cause [1,4]. Its estimated annual incidence is approximately 0.1 cases per 100,000 individuals and represents less than 1% of all space-occupying lesions within the spinal canal [5]. SSEH most frequently affects individuals in their fourth or fifth decade of life, with a mean age of 48 years and a slight male predominance, with a male-to-female ratio of 1.4:1 [1,6,7]. Our patient, a 35-year-old man, aligns with the male predominance reported in the literature, although he is slightly younger than the average age typically described [1]. This demographic detail highlights the variability in age at presentation and reinforces the need to maintain clinical suspicion even in patients outside the typical age range.

The etiology remains unclear in 30%-40% of patients, with no identifiable source of bleeding [5]. Our case aligns with this subgroup, as no specific bleeding source was found. This condition is most commonly attributed to the rupture of the posterior epidural venous plexus, a low-pressure vascular network susceptible to sudden increases in venous pressure, such as those occurring during Valsalva maneuvers [8,9]. In cases with rapidly progressive neurological deterioration, arterial rupture may also be implicated [7]. Reported risk factors include vascular malformations, coagulation disorders, anticoagulant therapy, and pregnancy. Nevertheless, 40%-60% of cases occur without any apparent predisposing factor [5], which is also consistent with our patient, who had no identifiable risk factors.

Neurological damage in SSEH results from a combination of mechanical compression and hemodynamic disturbances [10]. Early neurological deficits are primarily due to direct spinal cord compression, which leads to conduction block, demyelination, and rapid symptom progression [11]. Additionally, impaired venous outflow contributes to venous congestion and biochemical alterations, which result in white matter edema and axonal swelling, further contributing to progressive neurological deterioration [11-13]. 

Clinically, SSEH typically presents with sudden, localized pain at the site of hemorrhage, sometimes followed by a transient pain-free period before the onset of neurological deficits. This biphasic pattern has been well described in the literature and was observed in our patient [5]. The severity of neurological impairment depends on the level of spinal cord involvement and may include both motor and sensory deficits, most commonly presenting as paraparesis or quadriparesis. Although less frequent, hemiparesis can also occur and may mimic acute ischemic stroke [2,3,8]. In this case, the presence of hemiparesis prompted stroke activation; however, the absence of cranial nerve involvement and a preceding history of cervical pain raised clinical suspicion for a spinal etiology.

MRI is the diagnostic modality of choice, as it confirms the presence of an epidural hematoma and provides information about its age based on the temporal evolution of hemoglobin degradation products [14]. In this patient, MRI revealed an epidural hematoma extending from C3 to T2, spanning a longer spinal segment than typically reported, as most cases involve the C6-T3 levels [11]. The lesion appeared isointense on T1-weighted images, hyperintense on T2-weighted images, and hypointense on SWI, findings consistent with a transitional stage between the hyperacute and acute phases, which aligns with the reported clinical timeline [14].

Surgical decompression through laminectomy or hemilaminectomy with hematoma evacuation is the standard treatment for SSEH and is recommended within 48 hours to minimize the risk of permanent neurological damage [15]. In our case, a laminectomy from C3 to C7 was performed 32 hours after the onset of neurological deficits. The patient presented with incomplete deficits prior to surgery and continued physical therapy throughout the postoperative period. At the one-month follow-up, he demonstrated complete neurological recovery. This favorable outcome aligns with previous reports indicating that patients with incomplete deficits benefit most from timely intervention [16,17]. In contrast, complete deficits at presentation and delayed treatment are associated with significantly poorer outcomes [18].

## Conclusions

This case underscores the importance of considering SSEH in the differential diagnosis of sudden-onset hemiparesis, especially in the absence of cranial nerve involvement and when preceded by severe neck or back pain. Although SSEH is a rare condition, its clinical presentation can closely mimic that of an ischemic stroke, often leading to initial misdiagnosis and delays in appropriate treatment. Recognizing these key distinguishing features is essential for timely diagnosis and intervention, as early surgical decompression significantly improves the chances of neurological recovery and favorable clinical outcomes.
